# Identification and Quantification of Celery Allergens Using Fiber Optic Surface Plasmon Resonance PCR

**DOI:** 10.3390/s17081754

**Published:** 2017-07-31

**Authors:** Devin Daems, Bernd Peeters, Filip Delport, Tony Remans, Jeroen Lammertyn, Dragana Spasic

**Affiliations:** 1 KU Leuven—University of Leuven, BIOSYST-MeBioS, Willem de Croylaan 42, B-3001 Leuven, Belgium; devin.daems@kuleuven.be (D.D.); bernd.peeters@kuleuven.be (B.P.); filip.delport@kuleuven.be (F.D.); dragana.spasic@kuleuven.be (D.S.); 2 FOx Diagnostics NV—Veldstraat 120, B-9140 Temse, Belgium; 3 UHasselt—Hasselt University, Environmental Biology, Agoralaan Gebouw D, B-3590 Diepenbeek, Belgium; tony.remans@PXL.BE

**Keywords:** fiber optic SPR, food allergen, celery, qPCR, high-resolution melting

## Abstract

Accurate identification and quantification of allergens is key in healthcare, biotechnology and food quality and safety. Celery (*Apium graveolens*) is one of the most important elicitors of food allergic reactions in Europe. Currently, the golden standards to identify, quantify and discriminate celery in a biological sample are immunoassays and two-step molecular detection assays in which quantitative PCR (qPCR) is followed by a high-resolution melting analysis (HRM). In order to provide a DNA-based, rapid and simple detection method suitable for one-step quantification, a fiber optic PCR melting assay (FO-PCR-MA) was developed to determine different concentrations of celery DNA (1 pM–0.1 fM). The presented method is based on the hybridization and melting of DNA-coated gold nanoparticles to the FO sensor surface in the presence of the target gene (mannitol dehydrogenase, *Mtd*). The concept was not only able to reveal the presence of celery DNA, but also allowed for the cycle-to-cycle quantification of the target sequence through melting analysis. Furthermore, the developed bioassay was benchmarked against qPCR followed by HRM, showing excellent agreement (*R*^2^ = 0.96). In conclusion, this innovative and sensitive diagnostic test could further improve food quality control and thus have a large impact on allergen induced healthcare problems.

## 1. Introduction

Celery (*Apium graveolens*) is one of the most common foods causing allergic reactions in European countries, particularly in France, Switzerland and Germany. Notably, in France, 30% of anaphylactic reactions are caused by the ingestion of celery [1,2,3]. Although celery is consumed cooked or processed, compared to raw celery, food processing only lowers the allergenicity, but does not destroy the allergenic properties completely [2]. Therefore, celery was added to the list of fourteen groups of allergenic species with strict labelling legislation issued by the EU Commission (Directive 2003/89/EC and Directive 1169/2011) in order to ensure that consumers are adequately informed on the presence of allergenic ingredients in food [4,5].

The presence of food allergens, similar to food toxins and food pathogens, can have considerable consequences on food safety. Therefore, it is of the utmost importance to avoid and prevent accidental contaminations in the food industry. Accurate identification and quantification is key in this context. To achieve this, many food companies send a representative sample of their final products to a remote laboratory, since only a limited number of available tests can be performed on-site that are truly fast, affordable and easy-to-use.

The two main approaches to detecting food allergens, including in celery, are antibody-based immunoassays and the DNA-based polymerase chain reaction (PCR). At present, available ELISAs for the specific detection of celery show cross-reactivity with closely related members of the *Apiaceae* family, such as carrots [2,6,7] or parsley [8]. Therefore, several real-time (i.e., quantitative) PCR (qPCR) assays have been developed as a more specific alternative [9,10,11,12], targeting sequences from the *Api g 1* or the mannitol dehydrogenase (*Mtd*) gene. Although *Api g 1* codes for the major allergenic protein of celery, *Mtd* is more widely recommended for use in these assays due to the lower risk of cross-reactivity with sequences of other (closely) related species [10,13]. Despite their high specificity, qPCR assays are considered laborious and require sophisticated, bulky equipment, as well as trained personnel. Furthermore, qPCR and post-amplification high-resolution melting (HRM) analysis rely on costly fluorescent reporters for labelling DNA. All these intrinsic features make this method unsuitable for a simple on-site high-throughput detection of celery in food matrices [14].

Therefore, the aim of this paper was to develop a proof-of-concept innovative, fast, and highly sensitive bioassay on a compact fiber optic surface plasmon resonance (FO-SPR) biosensor platform. SPR is one of the most advanced label-free, real-time detection technologies that provides information not only for the quantification, but also on the kinetics, of the binding reaction [15,16]. Over the past years, a FO-SPR biosensor was developed as a cost-effective and easy-to-use alternative for the more expensive and complex SPR systems [17], such as the well-known commercially available Biacore instrument (GE Healthcare, Uppsala, Sweden). The FO-SPR biosensor makes use of multimode optical fibers that facilitate SPR generation and enable implementation of bioassays [18,19,20]. As has already been demonstrated by our group, the FO-SPR biosensor developed in-house has the unique capacity to quantify a variety of different molecules, ranging from proteins and nucleic acids to small molecules not only in the buffer but also in the complex sample matrices, such as whole blood, serum, plasma or milk [14,21,22,23,24]. Moreover, the platform is compatible with gold nanoparticles (Au NP), which are used for signal amplification and improvement of the bioassay sensitivity [25,26]. In our previous publication, a FO-SPR PCR melting assay (FO-PCR-MA) [14] was established for one-step cycle-to-cycle identification of multiple DNA targets on the same FO-SPR sensor. Here, the concept was further elaborated to enable quantification of celery DNA targets both in buffer and in cleaning water samples.

## 2. Materials and Methods

### 2.1. Reagents

All chemicals were of analytical reagent grade and were purchased from Sigma-Aldrich (Bornem, Belgium), unless stated otherwise. Citrate-stabilized Au NPs, with a mean diameter of 20 nm, were ordered from BBI international (Cardiff, UK). Celery (*Apium graveolens*) was bought from a local market. Two target regions were selected for celery detection (*Api* and *Mtd*). Target sequences reported in literature (*Api g 1* [27] and *Mtd* [10]), as well as target sequences selected in-house (*Mtd 2* and *Mtd 3*), sequences of primers used for DNA amplification and sequences of hybridization probes used in the FO-SPR bioassay are all presented in Table 1. The primers selected in-house were checked for the absence of homology with DNA sequences of other plants using the BLAST software (National Center for Biotechnology Information, Bethesda, MD, USA). Hybridization probes were modified with a 3′ C3 (probe 1) or 5′ C6 thiol modifier (-SH) (probe 2) for their immobilization on the FO-SPR sensor and the Au NPs gold surfaces, respectively. All the hybridization probes were equipped with a poly T spacer in order to improve the hybridization efficiency of the DNA [28]. The free 3′ end of the hybridization probe 2 was blocked from extension by the polymerase enzyme using a 3′ phosphate modification. The primers and hybridization probes were chemically synthesized by Integrated DNA Technologies (IDT, Haasrode, Belgium).

### 2.2. qPCR/HRM

A qPCR master mix (PerfeCTa Sybr Green Fastmix, QuantaBio, Beverly, CA, USA) was used, containing all necessary components for the qPCR reaction except for primers. The reaction mixture consisted of 6.50 µL PCR master mix, 0.87 µL of each primer (333 nM final concentration), 3.89 µL distilled H_2_O and 0.87 µL target DNA. The qPCR (using a Rotor-gene Q HRM from Qiagen, Venlo, The Netherlands) was optimized for standard cycling conditions with an initial 240 s enzyme activation step at 95 °C, followed by 30 cycles of 30 s annealing and extension at 60 °C and 5 s denaturation at 95 °C. Afterwards a HRM was conducted by increasing the temperature from 60 °C to 95 °C (ramp speed = 0.5 °C/s).

### 2.3. Surface Functionalization of FO-SPR Sensors and Au NPs with DNA

The FO-SPR instrument and accompanying sensors were manufactured as described by Knez et al. [14,21]. 5′ or 3′ thiol functionalized hybridization probes were first activated with dithiothreitol (DTT, 0.1 M in Phosphate Buffer (PB) 0.18 mM, pH 8.3). DTT was used to split thiol dimers which could prevent the surface functionalization process and it was removed from the solution by DNA purification with a NAP-5 Sephadex column (GE Healthcare, Oslo, Norway). Those 5′ or 3′ thiol functionalized hybridization probes were immobilized on the Au NPs or FO-SPR sensors by adding 1 µM of probes. An accelerated salt maturation protocol was used to maximize the DNA density on the Au NPs. [29] Au NPs were then washed three times in PB with 0.01% SDS and stored in the same buffer at 4 °C until further use. Both the FO-SPR sensors and Au NPs were backfilled to enable the PCR assays by incubating them in a 50 µM alkane thiol PEG (Polypure, Oslo, Norway) dissolved in pure ethanol for 2 h. Finally, both surfaces were washed three times with a 0.01% SDS PB buffer and stored afterwards at 4 °C in H_2_O until further use.

### 2.4. FO-PCR-MA in Solution

A PCR master mix (DimerEraser, Takara, Shiga, Japan) containing all needed components for the PCR reaction except for the primers was used. The reaction mixture was compiled with 50 µL PCR master mix, 3 µL of every primer (300 nM final concentration), 20 µL of functionalized Au NPs (1 nmol/L in distilled nuclease free H_2_O), 4 µL MgCl_2_ (50 mM), 4 µL NaCl (400 mM), 6 µL distilled H_2_O and 10 µL target DNA, unless stated otherwise. The reaction mixture was covered by a layer of mineral oil (Immobiline DryStrip Cover Fluid, GE healthcare, Diegem, Belgium) to protect it from evaporation during thermocycling. The FO-PCR-MA was optimized for standard cycling conditions, first with an enzyme activation step at 95 °C (30 s), followed by 40 cycles of 30 s annealing at 53 °C (ramp speed = 5.0 °C/s), 30 sec elongation at 72 °C (ramp speed = 5.0 °C/s), and finally 5 s denaturation at 90 °C (ramp speed = 1.0 °C/s).

### 2.5. Biological Sample Preparation

Three cleaning water samples (referred to as DNA 1–3) were spiked with 200 mg of celery (in 2 mL sample) from stem and leaf tissues, corresponding to the low pM range for stem tissue and the high fM range for leaf tissue. DNA was extracted from these samples using the commercial Qiagen DNeasy plant mini kit (Venlo, The Netherlands). The total DNA amount was measured by Qubit dsDNA HS Assay Kit (Thermo Fisher Scientific, Gent, Belgium). These spiked DNA 1–3 samples were evaluated both with qPCR and FO-PCR-MA for the presence of celery.

### 2.6. Data Processing

qPCR/HRM was recorded with the supplied program of Rotor-gene Q HRM and analyzed with the supplied Rotor-gene Q series Software version 2.3.1 (Qiagen, Venlo, The Netherlands). The data analysis (first derivative dF/dt and the threshold) was automatically calculated by this program.

FO-PCR-MA data acquisition was done with the in-house developed LabView program (National Instruments, Austin, TX, USA) to control two spectrometers and the NiDaq coupled thermocouples. By combining the SPR and thermocouple data, a first order derivative could be calculated for each PCR melting cycle. The resulting melting peak was fitted in Matlab (MathWorks, Natick, MA, USA) using a Gaussian fit to determine the T_m_ and evaluate the melting peak shape for each PCR cycle. The threshold was calculated as x_NTC_ + 3 σ_noise_, where x_NTC_ is the average value of the non-template control (*n* = 40 cycles) and σ_noise_ equals one standard deviation.

The intraclass correlation coefficient (ICC) [30], which assesses the reliability of ratings by comparing the variability of different ratings of the same object, was calculated with the statistical package R (version 2.11.1, R foundation for Statistical Computing, Vienna, Austria).

## 3. Results

### 3.1. Selection of Celery Target Sequence Using qPCR/HRM Analysis

qPCR followed by HRM analysis is the most commonly used method for detection of celery [9,10,11,12]. In general, HRM analysis is performed on double-stranded PCR-amplified DNA samples by heating the DNA between 60 °C and 95 °C. At the point known to be the melting temperature (T_m_) of the DNA target, two strands of DNA are separated, which is accompanied by a drop in fluorescent signal. This change in signal is due to the release of intercalating fluorescent dyes that have the unique properties of selectively binding only to double-stranded DNA and brightly fluorescing when in this bound state. Because the T_m_ is highly dependent on the DNA sequence, even two sequences of the same length, differing in only one nucleotide, will have different T_m_ values. Therefore, HRM is used for detecting mutations, polymorphisms and epigenetic differences, but also for target identification, the latter being of relevance for this study (Supplementary Information Figure S1). qPCR/HRM was, for these reasons, selected here as a reference technology.

**Figure 1:**
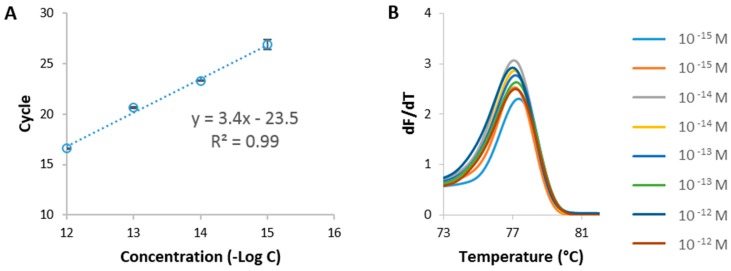
qPCR followed by HRM analysis, using the *Mtd 3* sequence. (**A**) Calibration curve obtained by plotting the amount of cycles needed to reach the detection threshold in function of the concentrations (semi-log plot). Error bars represent one standard deviation (*n* = 2); (**B**) Melting analysis of the *Mtd 3* target after 30 qPCR cycles at concentrations ranging from 1 pM to 1 fM.

qPCR/HRM was performed first to choose the most appropriate region from the celery genome to use for its detection (Supplementary Information Figure S2). Synthetic DNA targets were designed based on 4 different sequences from the two celery genes of interest, namely *Api g 1* and *Mtd* (Table 1) and used as targets in the qPCR/HRM analysis. One sequence, here referred to as *Mtd 3*, was identified as the most optimal one due to the absence of signal in the non-template control (NTC). *Mtd 3* corresponds to a 109 bp region of the celery sequence in the *Mtd* gene. Using *Mtd 3* synthetic DNA at various concentrations, ranging from 1 pM to 1 fM, a calibration curve was obtained as depicted in Figure 1A. This calibration curve will be further used to benchmark the sensitivity of the established FO-PCR-MA, as well as to determine the celery concentration in biological samples. The T_m_ of the selected target (77.1 ± 0.2 °C) was determined based on the HRM profile following the qPCR reaction (Figure 1B) (*n* = 8, 2 repetitions of 4 different concentrations). The assessed T_m_ will give information concerning the identification of the amplified target sequence.

### 3.2. Establishing FO-PCR-MA for Celery DNA Detection

**Figure 2:**
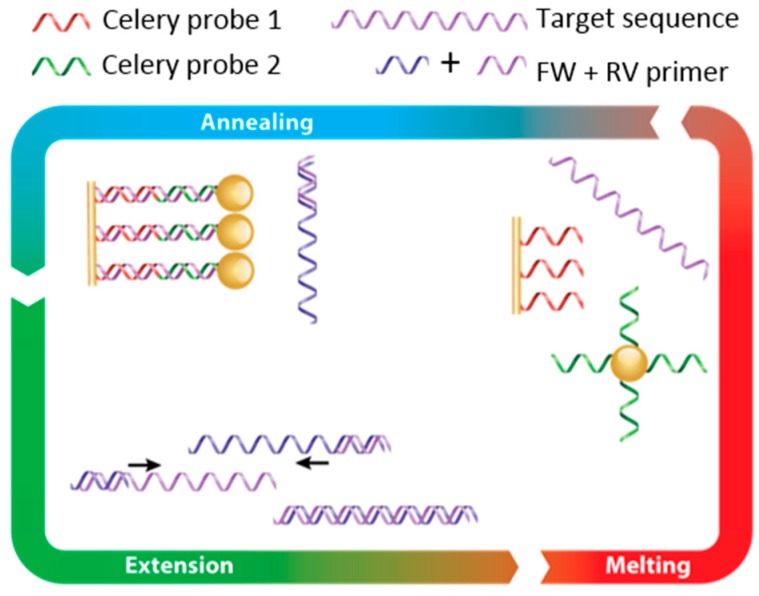
Conceptual overview of the FO-PCR-MA. The PCR primers specific for celery targets are used to amplify a target region during a standard PCR reaction. The amplicons hybridize during the PCR reaction to complementary DNA probes on the FO-SPR sensor and those on Au NPs. During the normal PCR thermocycling, used to denature the amplicons, the FO-SPR sensor registers a change in mass (i.e., change in refractive index), which can be used for determining characteristic T_m_ of the target sequence and thus its identification.

In order to develop a proof-of-concept fast and highly sensitive FO-SPR bioassay for celery DNA detection, we started from the FO-PCR-MA concept previously established in the group for bacterial detection [14]. The FO-PCR-MA concept is schematically illustrated in Figure 2. Briefly, DNA probe 1 for celery DNA detection is immobilized on the FO-SPR sensor surface, and DNA probe 2 is present on the Au NPs, each of them designed to be complementary to the first and second half of the target celery sequence, respectively. In the presence of the target DNA, these two probes hybridize to the target, thereby bringing the Au NPs very close to the sensor surface. During the PCR, the target DNA is amplified, resulting in an increasing amount of DNA binding to the sensor (indicated as hybridization on the scheme). During the heating phase (indicated as melting on the scheme), the target DNA strands disconnect from the probes on the FO-SPR and Au NPs, releasing Au NPs from the FO-SPR surface. This causes a change in mass on the sensor and consequently a change in refractive index, which can be used for determining the characteristic T_m_ of the target sequence and thus its identification.

**Figure 3:**
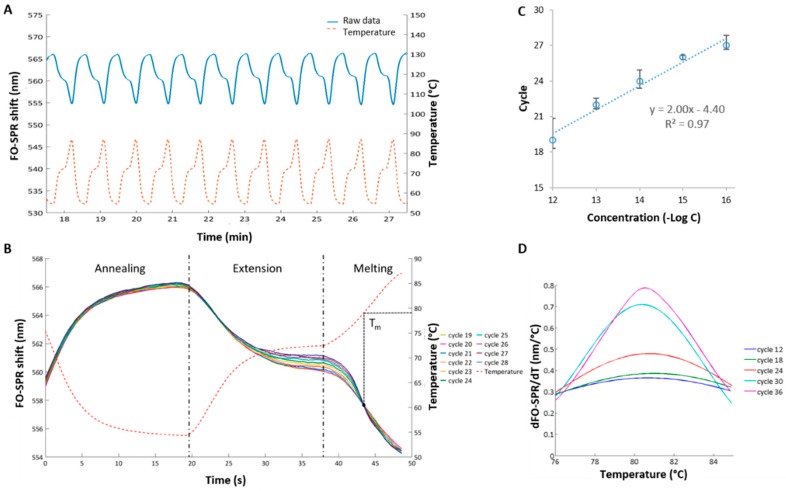
Quantification of celery *Mtd 3* target using FO-PCR-MA. (**A**) Raw data of an FO-SPR PCR measurement. One measurement channel measures changes in refractive index due to the thermocycling. The FO-SPR signal is the inverse of the temperature measured with a thermocouple; (**B**) FO-SPR signal for 10 PCR cycles containing 100 fM celery DNA. Initially the FO-SPR signal is the exact inverse of the temperature signal; however, as the DNA concentration reaches the detection threshold of the FO-SPR biosensor, a melting point is visible around 44 s; (**C**) Calibration curve obtained by plotting the number of cycles, needed to reach the detection threshold, as a function of the target concentration (semi-log plot). Error bars represent the standard error on the last 5 melting peak signals of FO-PCR-MA cycles needed to reach the detection threshold (see Supplementary Information); (**D**) First order derivative of the FO-SPR signal to the temperature, which allows resolving the melting point of 100 fM target DNA (*Mtd 3*) amplified with the PCR on the FO-SPR device. Once the cycle-to-cycle amplification of the DNA target during the FO-PCR-MA reaction has reached the detection threshold of the FO-SPR sensor, the dSPR/dt signal increases every PCR round.

Although the established FO-PCR-MA was demonstrated for its multiplexing capacity, monitoring of the amplification reaction in real-time and detecting multiple mutations in the target sequence [14], the real sensitivity of this assay and its limit of detection have not been explored. Therefore, starting from the known concept, a new FO-PCR-MA was developed here for the celery detection. A synthetic celery target, indicated as *Mtd 3*, was used together with specific DNA probes 1 and 2, as well as specific primers, as depicted in Table 1. The cycle-to-cycle amplification of celery DNA in the solution was studied in real-time in the presence of the FO-SPR sensor. Initially, the FO-SPR signal of the FO-PCR-MA reaction, which was continuously monitored with a thermocouple (Figure 3A), shows to be the inverse of the temperature change. However, from the moment the DNA target is amplified above the FO-PCR-MA signal detection threshold of the FO-SPR, the melting signal of the amplified DNA target will superimpose on the refraction index shift of the temperature (Figure 3B) [14]. A calibration curve was obtained with concentrations of synthetic DNA ranging from 1 pM to 0.1 fM (Figure 3C), which was one order of magnitude lower than the qPCR calibration curve. Moreover, melting analysis performed simultaneously during the PCR reaction on the FO-SPR revealed a T_m_ of 79.4 ± 0.5 °C for the *Mtd 3* target (Figure 3D). The determined T_m_ is essential for the specific identification of amplified sequences in order to distinguish them from the non-specific amplification that can occur. The FO-PCR-MA signal threshold of 0.095 nm was calculated as described in Section 2.6 and used to determine the exceeding cycle for each concentration (Figure S3, Supplementary Information). By taking the first order derivative of the FO-SPR signal with respect to the temperature, the T_m_ of the celery DNA target was resolved very precisely and corresponded well to the T_m_ value determined using the reference qPCR/HRM technology (Figure 1B). The small difference in T_m_ values can be attributed to the different PCR reaction mixtures used in two assays as it has been shown previously that buffer ionic strengths can have a strong influence on the T_m_ [14].

In conclusion, the performance of the FO-PCR-MA was slightly better than the qPCR concerning the detection range, but in accordance with the HRM in terms of its variability and the estimation of the T_m_. The obtained FO-PCR-MA calibration curve will be used further to determine the celery DNA concentration in biological samples, while the assessed T_m_ will be used to confirm the identification of the amplified target sequence. Moreover, the newly developed FO-PCR-MA combines PCR and HRM in one step, which reduces the overall assay time for at least 25 min, whereas the time needed to perform sample preparation is the same for both approaches. Here, the exact difference in time-to-result was 1460 s, or 24.3 min, as the qPCR (30 cycles) followed by HRM took 4810 s while the FO-PCR-MA (40 cycles) had a time-to-result of 3350 s. Furthermore, as FO-PCR-MA provides cycle-to-cycle information during the melting assay in real time, the reaction can be stopped earlier. This means that, from the moment the signal reaches the threshold, all the information concerning identification and quantification of the sample will be present, which can significantly reduce the assay time.

### 3.3. Validation of Established FO-PCR-MA with Biological Samples

**Figure 4:**
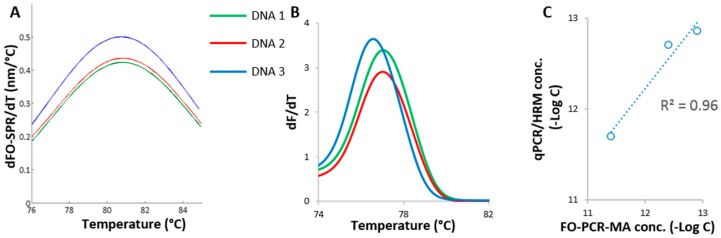
Detection of celery in biological samples. (**A**) FO-PCR-MA of the *Mtd 3* target sequence in sample DNA 1, DNA 2 and DNA 3, cycle 35; (**B**) HRM results of sample DNA 1, DNA 2 and DNA 3 after qPCR; (**C**) Comparison of the *Mtd 3* levels determined with the in-house developed FO-PCR-MA biosensor (40 cycles) and the qPCR/HRM test (30 cycles).

The developed FO-PCR-MA biosensor for the quantification of celery DNA was further benchmarked with qPCR/HRM using three cleaning water samples spiked with celery. These samples were prepared as mentioned in Section 2.5. Sample DNA 1 and DNA 2 were spiked with celery derived from stem tissue, while DNA 3 contained celery leaf tissue. The celery DNA concentrations were determined in these samples with FO-PCR-MA methodology (Figure 4A), using the calibration curve shown in Figure 3C and with the qPCR/HRM technology (Figure 4B), using the calibration curve shown in Figure 1A. The same FO-PCR-MA signal threshold of 0.095 nm was applied on the spiked celery samples as it was for the samples in the calibration curve (Figure S4, Supplementary Information). Based on the qPCR/HRM and the FO-PCR-MA, the T_m_ of the three samples was estimated at 76.9 ± 0.3 and 80.1 ± 0.5 °C, respectively (*n* = 3 for both analysis). The obtained values were in perfect accordance with the T_m_ determined in Figure 1B and Figure 3D, proving the correct identification of the amplified *Mtd 3* target sequence in the cleaning water samples. As shown in Figure 4C, the celery concentrations quantified with the FO-PCR-MA biosensor correlated linearly with the corresponding qPCR/HRM results (*R*^2^ = 0.96). Moreover, the ICC was calculated to be 0.94 (*n* = 3).

## 4. Conclusions

For some hypersensitive patients, even traces of celery in foods can often elicit life-threatening allergic reactions. Therefore, accurate identification and quantification of this common allergen is key in healthcare, biotechnology, and food quality and safety. The specificity issues when detecting celery DNA have been partially resolved by the introduction of qPCR/HRM. In search of new techniques to quantify celery, a FO-SPR biosensor developed in-house was explored in this proof-of-concept paper. Here, a real-time FO-PCR-MA approach was established based on the *Mtd* gene of celery and used to quantify this target between 1 pM and 0.1 fM. The developed concept was comparable with the qPCR/HRM analysis in terms of the linear range and the determination of the melting peaks. Finally, the FO-PCR-MA was benchmarked with a reference qPCR/HRM technique using cleaning water samples spiked with celery, showing excellent agreement, as confirmed with the ICC of 0.94. In conclusion, this non-fluorescence-based technology platform holds great potential for the real-time detection and quantification of celery allergens. Furthermore, as this platform is fast, cost-effective and automated, it fulfils the requirements for the next generation of diagnostic tests.

## Figures and Tables

**Table 1:** Overview of used oligonucleotides for the qPCR and the FO-PCR-MA.

Targets (5′ → 3′)	Sequence
*Api g 1* (76-mer)	GGG CTT TGT CAT TGA TGT TGA CAC AGT CCT TCC CAA GGC TGC GCC TGG AGC TTA CAA GAG TGT CGA AAT CAA GGG A
*Mtd* (101-mer)	CGA TGA GCG TGT ACT GAG TCA GTG TTA TGT TTG GAT TAC GGT GTG ATG AGT CAG CGT TAT CTG TTT TTA TAT GTT TGG TAT GAT TAA TGT TAG TTC CTA TT
*Mtd 2* (101-mer)	CCT TGT TAG CGG AGT CTA AAT CGG AAT CTA AAT CTA AAT CAT TTT AAG CAT GTT AGC CCT TGT ATT TTG GCT TTT GGC TTT TAA CCA TTT TGT TC
*Mtd 3* (109-mer)	CCC GTA CGA GAT ATA TTT TTG TCT GGT TTG AGA TAT ATA TTA CAT GCT GAG TCA CGA TGA GCG TGT ACT GAG TCA GTG TTA TGT TTG GAT TAC GGT GTG ATG AGT CAG C
Primers (5′ → 3′)	
*Api g 1* forward (22-mer)	GGG CTT TGT CAT TGA TGT TGA C
*Api g 1* reverse (24-mer)	TCC CTT GAT TTC GAC ACT CTT GTA
*Mtd* forward (20-mer)	CGA TGA GCG TGT ACT GAG TC
*Mtd* reverse (29-mer)	AAT AGG AAC TAA CAT TAA TCA TAC CAA AC
*Mtd 2* forward (24-mer)	CCT TGT TAG CGG AGT CTA AAT CGG
*Mtd 2* reverse (20-mer)	GAA CAA AAT GGT TAA AAG CC
*Mtd 3* forward (26-mer)	CCC GTA CGA GAT ATA TTT TTG TCT GG
*Mtd 3* reverse (23-mer)	GCT GAC TCA TCA CAC CGT AAT CC
Hybridization probes (5′ → 3′)	
*Mtd 3* probe 1 (66-mer)	GTG ACT CAG CAT GTA ATA TAT ATC TCA AAC CAG ACA AAA ATA TAT CTC GTA CGG GTT TTT TTT TT
*Mtd 3* probe 2 (64-mer)	TTT TTT TTT TGC TGA CTC ATC ACA CCG TAA TCC AAA CAT AAC ACT GAC TCA GTA CAC GCT CAT C

## Supplementary Materials

The following are available online at www.mdpi.com/1424-8220/17/8/1754/s1, Figure S1: A multiplex FO-SPR melting analysis performed for carrot DNA and celery DNA target sequences on the same FO-SPR sensor, Figure S2: qPCR signal for different concentrations of different synthetic targets (1 nM–10 fM): *Api g 1* (A), *Mtd* (B), *Mtd 2* (C) and *Mtd 3* region (D). Non template controls (NTC) have been included. Figure S3: Presentation of the melting peak signals in function of the number of PCR cycles. The FO-PCR-MA signal threshold of 0.095 nm is depicted with the red line on the figures. Analysis of the signal height for different DNA concentrations (10^−12^–10^−16^ M) is shown for the *Mtd 3* target sequence. Furthermore, the non-template control (NTC) analysis of the signal height is presented in the bottom right corner of the figure, Figure S4: Presentation of the melting peak signals in function of the number of PCR cycles. The FO-PCR-MA signal threshold of 0.095 nm is shown on the figures. Analysis of the signal height of different spiked biological samples (DNA 1, DNA 2 and DNA 3) are shown for the *Mtd 3* target sequence.
