# Supplementary material for: Identification and Quantification of Celery Allergens Using Fiber Optic Surface Plasmon Resonance PCR
Source: Sensors (Basel). 2017 Jul 31;17(8):1754. doi: 10.3390/s17081754 (PMC5579924; doi:10.3390/s17081754)
Supplement: Supplementary file 1 [file sensors-17-01754-s001.pdf]

Identification and Quantification of Celery Allergens Using Fiber Optic Surface Plasmon Resonance PCR

Devin Daems [1], Bernd Peeters [1], Filip Delport [1,2], Tony Remans [3], Jeroen Lammertyn [1,2,*] and Dragana Spasic [1]

[1] KU Leuven—University of Leuven, BIOSYST-MeBioS, Willem de Croylaan 42, B-3001 Leuven, Belgium. devin.daems@kuleuven.be (D.D.); bernd.peeters@kuleuven.be (B.P.); filip.delport@kuleuven.be (F.D.); dragana.spasic@kuleuven.be (D.S.)
[2] FOx Diagnostics NV—Veldstraat 120, B-9140 Temse, Belgium
[3] UHasselt—Hasselt University, Environmental Biology, Agoralaan Gebouw D, B-3590 Diepenbeek, Belgium; tony.remans@PXL.BE
* Correspondence: jeroen.lammertyn@kuleuven.be; Tel.: +32-16321459

Supplementary Information

The specificity of a FO-SPR melting assay, as described by Knez et al. [1,2], was tested by performing multiplex analysis of celery DNA sequence (originating from *Api g 1* celery gene) and carrot DNA sequence (originating from dau c1 carrot gene). Carrot DNA sequence was included as exogenous positive control to demonstrate the specificity of our assay because it is well known from literature that carrots show high cross reactivity with celery [3–5]. 15 µL of the DNA targets (10 nM final concentration) was added to 42 µL PBS with 300 mM NaCl, and 43 µL functionalized gold nanoparticles. Before each measurement, the samples were heated to 94 °C for 5 s. Subsequently, the temperature was lowered to allow hybridization. After 15 min, the temperature was slowly raised (0.1 °C/s) till 94 °C. The results of the FO-SPR melting analysis experiments revealed 15 °C difference in melting temperature between the carrot DNA and the celery DNA (Figure S1), confirming indeed that the designed FO-SPR melting assay is specific.

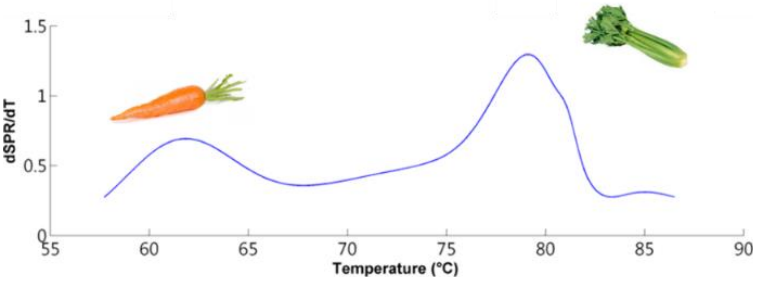

Figure S1. A multiplex FO-SPR melting analysis performed for carrot DNA and celery DNA target sequences on the same FO-SPR sensor.

Nevertheless, when the same celery sequence from *Api g 1* gene was tested using qPCR/HRM together with 3 other sequences from *Mtd* celery gene (*Mtd*, *Mtd 2* and *Mtd 3*), *Mtd* (Figure S2B) and *Mtd 3* (Figure S2D), were identified as more optimal compared to *Api g 1* due to the reproducible absence of signal in the non-template control (NTC) after 30 cycles (Figure S2). Because the specificity of *Mtd 3* sequence was confirmed through BLAST report, this sequence was finally selected for all experiments as presented in the manuscript.

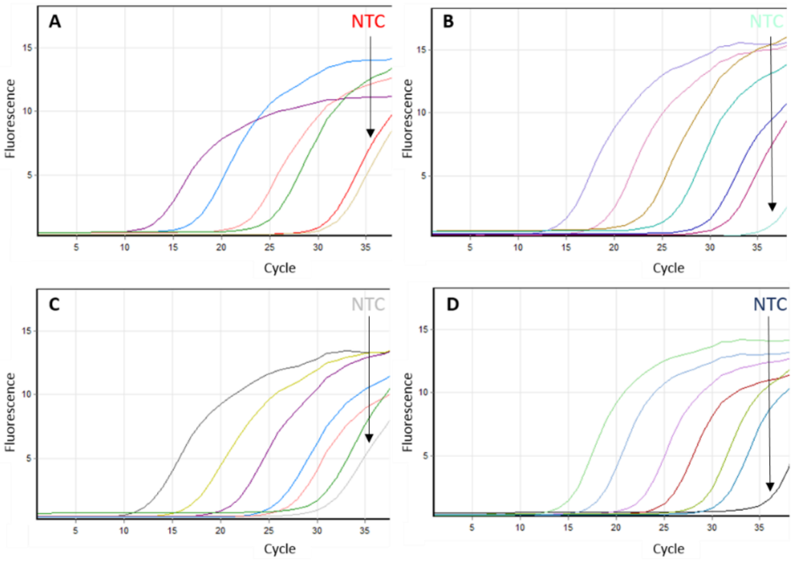

Figure S2. qPCR signal for different concentrations of different synthetic targets (1 nM–10 fM): *Api g 1* (A), *Mtd* (B), *Mtd 2* (C) and *Mtd3* region (D). Non template controls (NTC) have been included.

The amplification of DNA in solution, by heating up and cooling down the PCR mixture, was studied in the presence of the FO-SPR sensor. The FO-SPR signal is continuously monitored with a thermocouple, which is the inverse of the temperature change. However, from the moment DNA target is amplified through FO-PCR-MA above the detection threshold of the FO-SPR, the melting signal of the amplified DNA target will superimpose on the refraction index shift of the temperature measured with the FO-SPR sensor [6]. By taking the first order derivative of the SPR signal with respect to the temperature, the Tm of the *Mtd 3* target was resolved very precisely. When the FO-SPR melting peak height is plotted versus the PCR cycle number for different *Mtd 3* concentrations (Figure S3), it can be seen that melting peaks increase cycle-to-cycle. In Figure S4, the melting peak signals of the three spiked samples are visualized in function of the number of PCR cycles.

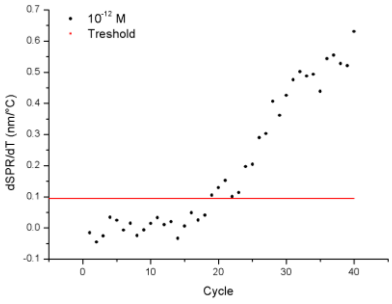

(**a**)

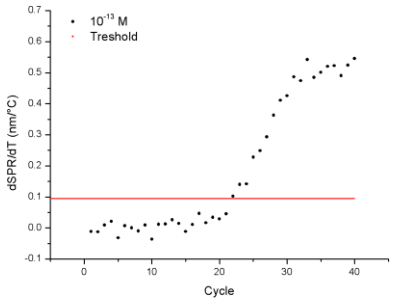

(**b**)

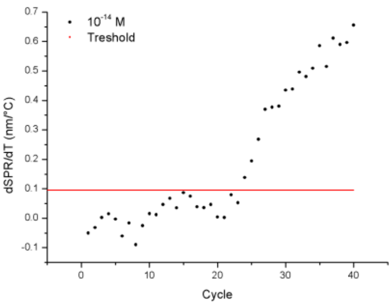

(**c**)

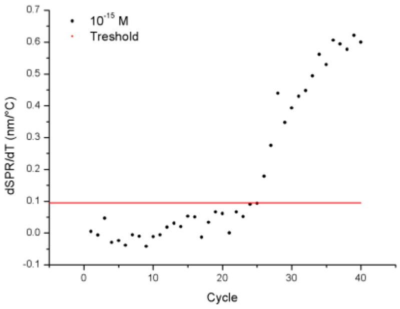

(**d**)

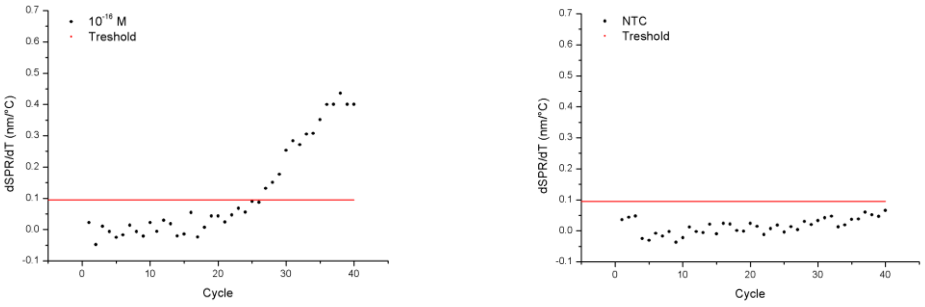

(**e**) (**f**)

Figure S3. Presentation of the melting peak signals in function of the number of PCR cycles. The FO-PCR-MA signal threshold of 0.095 nm is depicted with the red line on the figures. Analysis of the signal height for different DNA concentrations (10^{-12}–10^{-16} M) is shown for the *Mtd 3* target sequence. Furthermore, the non-template control (NTC) analysis of the signal height is presented in the bottom right corner of the figure.

To determine the error bars in Figure 3C, the following procedure was followed. First, the standard deviation on the FO-SPR signal of the last 5 cycles (here: cycles 36 till 40), where saturation occurs, was calculated. Assuming that the standard deviation is constant and independent of the cycle number, this value was transferred to the FO-PCR-MA signal threshold value (Th). The FO-PCR-MA signal threshold value plus one standard deviation (Th + 1 sigma) and minus one standard deviation (Th − 1 sigma) were calculated. The variability on the cycle number was then determined by taking the highest cycle number for which the FO-SPR signal was equal to Th − 1 sigma and by taking the lowest cycle number for which the FO-SPR value was equal to Th + 1 sigma. As this conversion is a non-linear transformation, the error bars in Figure 3C are typically asymmetric.

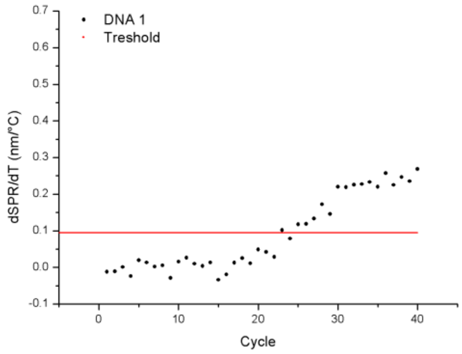

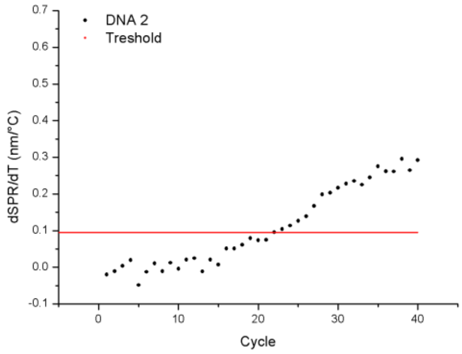

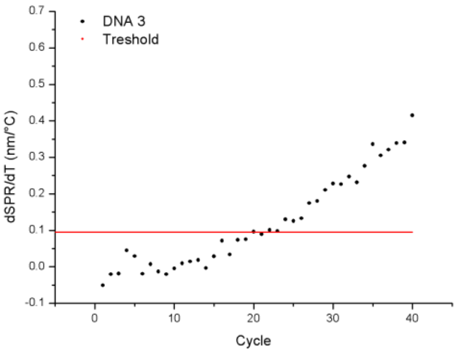

Figure S4. Presentation of the melting peak signals in function of the number of PCR cycles. The FO-PCR-MA signal threshold of 0.095 nm is shown on the figures. Analysis of the signal height of different spiked biological samples (DNA 1, DNA 2 and DNA 3) are shown for the *Mtd 3* target sequence.

References

1. Knez, K.; Janssen, K.P.F.; Pollet, J.; Spasic, D.; Lammertyn, J. Fiber-optic high-resolution genetic screening using gold-labeled gene probes. *Small* **2012,** *8,* 868–872.
2. Knez, K.; Janssen, K.P.F.; Spasic, D.; Declerck, P.; Vanysacker, L.; Denis, C.; Tran, D.T.; Lammertyn, J. Spherical nucleic acid enhanced FO-SPR DNA melting for detection of mutations in legionella pneumophila. *Anal. Chem.* **2013**, *85,* 1734–1742.
3. Fuchs, M.; Cichna-Markl, M.; Hochegger, R. Development and validation of a duplex real-time PCR method for the simultaneous detection of celery and white mustard in food. *Food Chem.* **2013**, *141,* 229–235.
4. Fste, C.K.; Jonscher, K.R.; Slit, L. Klawitter, J.; Lvberg, K.E.; Moen, L.H. Differentiating Cross-Reacting Allergens in the Immunological Analysis of Celery (*Apium graveolens*) by mass spectrometry. *J. AOAC Int.* **2010**, *93*, 451–461.
5. Sun, M.; Gao, H.; Xiao, X.; Chen, J.; Liu, C.; Feng, L. A novel loop-mediated isothermal amplification method for detection of the carrot materials in food, *Eur. Food Res. Technol.* **2015**, *214,* 295–302.
6. Daems, D.; Knez, K.; Delport, F.; Spasic, D.; Lammertyn, J. Real-time PCR melting analysis with fiber optic SPR enables multiplex DNA identification of bacteria, *Analyst* **2016**, *141,* 1906–1911.
